# Functional Heterogeneity of Embryonic Stem Cells Revealed through Translational Amplification of an Early Endodermal Transcript

**DOI:** 10.1371/journal.pbio.1000379

**Published:** 2010-05-25

**Authors:** Maurice A. Canham, Alexei A. Sharov, Minoru S. H. Ko, Joshua M. Brickman

**Affiliations:** 1 Institute for Stem Cell Research, Medical Research Council – Centre for Regenerative Medicine, School of Biological Sciences, University of Edinburgh, Edinburgh, United Kingdom; 2 Laboratory of Genetics, National Institute on Aging, National Institutes of Health Biomedical Research Center, Baltimore, Maryland, United States of America; Osaka University, Japan

## Abstract

ES cells are defined as self-renewing, pluripotent cell lines derived from early embryos. Cultures of ES cells are also characterized by the expression of certain markers thought to represent the pluripotent state. However, despite the widespread expression of key markers such as Oct4 and the appearance of a characteristic undifferentiated morphology, functional ES cells may represent only a small fraction of the cultures grown under self-renewing conditions. Thus phenotypically “undifferentiated” cells may consist of a heterogeneous population of functionally distinct cell types. Here we use a transgenic allele designed to detect low level transcription in the primitive endoderm lineage as a tool to identify an immediate early endoderm-like ES cell state. This reporter employs a tandem array of internal ribosomal entry sites to drive translation of an enhanced Yellow Fluorescent Protein (Venus) from the transcript that normally encodes for the early endodermal marker Hex. Expression of this Venus transgene reports on single cells with low Hex transcript levels and reveals the existence of distinct populations of Oct4 positive undifferentiated ES cells. One of these cells types, characterized by both the expression of the Venus transgene and the ES cells marker SSEA-1 (V^+^S^+^), appears to represent an early step in primitive endoderm specification. We show that the fraction of cells present within this state is influenced by factors that both promote and suppress primitive endoderm differentiation, but conditions that support ES cell self-renewal prevent their progression into differentiation and support an equilibrium between this state and at least one other that resembles the Nanog positive inner cell mass of the mammalian blastocysts. Interestingly, while these subpopulations are equivalently and clonally interconvertible under self-renewing conditions, when induced to differentiate both in vivo and in vitro they exhibit different behaviours. Most strikingly when introduced back into morulae or blastocysts, the V^+^S^+^ population is not effective at contributing to the epiblast and can contribute to the extra-embryonic visceral and parietal endoderm, while the V^−^S^+^ population generates high contribution chimeras. Taken together our data support a model in which ES cell culture has trapped a set of interconvertible cell states reminiscent of the early stages in blastocyst differentiation that may exist only transiently in the early embryo.

## Abstract 2

Detection of low-level, lineage-specific transcription aids in the identification of lineage-primed populations of ES cells provides a new framework for pluripotency.

## Author Summary

Embryonic stem (ES) cells are karyotypically normal, embryo-derived cell lines that are pluripotent, i.e. capable of generating all the cell types of the future organism, but not the extra-embryonic lineages. What gives ES cells this unique capacity? Here, we use a fluorescent reporter cell line that employs translational amplification to visualize single ES cells expressing low levels of lineage-specific genes. With this reporter we split ES cell cultures into two fractions that both express certain stem cell markers but only one of which expresses low levels of an endodermal marker gene. Following purification, single cells from either fraction are equally competent to re-establish a heterogeneous culture. However, when challenged to differentiate immediately after purification, each exhibits strong lineage bias, with the endoderm marker-expressing fraction unexpectedly able to contribute to the extra-embryonic endoderm in chimeric embryos. These data suggest that ES cells expand under steady-state conditions as a heterogeneous mix of lineage-biased—but not lineage-committed—cell types. We propose that these observed uncommitted substates exist temporarily in vivo, but are perpetuated in vitro under the selectively self-renewing conditions of ES cell culture. Our findings suggest that pluripotency is determined by the capacity of a mixed population of lineage-biased intermediates to commit to different cell fates in specific contexts.

## Introduction

ES cells are an in vitro cell line derived from the inner cell mass (ICM) of the early mammalian blastocyst [1],[2]. In mouse they are defined functionally as a karyotypically normal immortal cell line that can give rise to all the future lineages of the conceptus [3]. Thus they can self-renew indefinitely and continually generate progeny with equivalent pluripotent properties. The pluripotent properties of ES cells can be demonstrated by in vitro differentiation or by reintroduction of these cells back into chimeric embryos by blastocyst injection or morula aggregation.

ES cells can be described based on a characteristic morphology, the presence of cell surface markers such as SSEA-1 and Pecam1, or the expression of the key transcription factors such as Oct4, Sox2, Nanog, and a number of ES cell-specific transcripts (ECATs) [4]–[6]. However, while these markers are useful tools, ES cells can only be defined based on retrospective function. A culture can be said to contain ES cells, if a chimera generated from the injection of these cells contains “ES cell derived,” somatic, and in particular, germ line tissue. Interestingly, attempts to define the number of founder ES cells in chimera experiments suggest that most somatic tissues are formed from one or two of the 10–15 cells injected into a typical blastocyst [7]. Thus despite indistinguishable morphology and apparent homogenous expression of pluripotent markers such as Oct4, functional ES cells may represent only a small component of any ES cell culture.

Recent observations suggest that there may be lineage-specific markers expressed in sub-populations of ES cell cultures. In particular, the expression of the ICM markers Nanog, Rex1, and Stella has been shown to be heterogeneous [8]–[12]. Does this heterogeneity define a functional subpopulation of cells in ES cell cultures? While levels of Nanog can affect the propensity to differentiate, Nanog^−/−^ ES cells are able to contribute to all lineages of the conceptus with the exception of the germ cells [8]. Moreover, all of these studies compare the pluripotent potential of the marked ICM-like population to mixed fractions that are considered a single further differentiated intermediate cell type. Interestingly, while not linked to Nanog, the somite segmentation clock gene Hes1 also displays heterogeneous expression that is related to periodic oscillations and differential rates of differentiation [13].

ES cells are derived from a stage of development in which key early lineage specification events are occurring. ICM cells are formed from the inner cells of the morula as the outer cells form the first extra-embryonic or trophoblast lineage. A day later, at implantation (4.5 *dpc.*), the ICM then gives rise to two lineages, primitive ectoderm (PrEc or epiblast) and primitive endoderm (PrEn). The epiblast is the source of all embryonic tissue and the PrEn the source of both extra-embryonic endoderm lineages, visceral and parietal. Although the visceral endoderm (VE) itself does not contribute to the embryo proper, an important early embryonic signalling centre is formed in VE at the embryo's distal tip and these cells will then migrate anteriorly to form the anterior visceral endoderm (AVE) [14]–[16].

When injected into host blastocysts, cells derived directly from the ICM of an expanded blastocyst stage can contribute to the PrEn as well as the fetus [17],[18]. However, cells derived from the early epiblast are only able to contribute to embryonic lineages and not those derived from the PrEn [18]–[20], while PrEn cells can only contribute to their own lineage by colonizing the visceral and mostly parietal endoderm in chimera experiments [20]–[22]. While ES cells are derived from the ICM, they predominantly contribute to embryonic lineages. This notion, that ES cells can contribute only to the somatic lineages, has been exploited for the study of embryonic versus extra-embryonic phenotypes [14] and is the reason they are defined as pluripotent, rather than totipotent. However, despite this consensus view there is some evidence from blastocyst injection that ES cells can colonize the yolk sac descendants of the PrEn [23]. In vitro, ES cells can generate PrEn-like cells either in response to LIF withdrawal [24] or through forced expression of the transcription factors Gata4 or Gata6 [25],[26]. ES cell cultures also express low levels of Gata4 and Gata6, suggesting the presence of either background levels of PrEn gene expression or basal levels of PrEn differentiation [25],[27].

One of the earliest markers of anterior asymmetry in the AVE is the homeobox transcription factor Hex. While Hex is discretely expressed in the VE on the anterior side of the embryo, it is initially expressed throughout the early PrEn [28] and like the GATA factors, Hex transcripts are also detectable in some ES cell cultures [29]. However, the levels of this transcript are presumably extremely low as they were not detected in fluorescent Hex reporter ES cell lines [30]. Here we explore the significance of this low transcript level and ask what it represents in ES cell culture. We use an ES cell line in which low levels of Hex transcript are visualized based on the expression of the enhanced YFP, Venus coupled to a unique translational amplifier. Using this cell line we show that apparently undifferentiated ES cell cultures consist of at least three cell types defined by this lineage-specific low-level transcription and the expression of the ES cell markers Oct4 and Nanog. Venus positive cells experiencing low-level transcription at the Hex locus, but still expressing the ES cell markers SSEA-1 and Oct4, show elevated levels of PrEn gene expression and reduced levels of early ICM markers such as Nanog. This early PrEn state does not appear to represent differentiation but rather exists in equilibrium with the Venus negative cell states. Manipulation of either FGF signalling or Nanog expression levels can alter the ratio of cell types present in this state and single Venus positive or negative cells can regenerate this equilibrium with apparently identical kinetics under self-renewing conditions. However, when ES cells are purified based on expression of this Venus allele and the ES cell marker SSEA-1, and then followed in differentiation either in vivo or in vitro, the two populations of ES cells have very different properties. The Venus negative population contributes efficiently to the epiblast in chimeras and remains in the centre of differentiating embryoid bodies (EBs). The Venus positive population does not efficiently contribute to somatic lineages, appears at the outside of EBs, and has the capacity to colonize the visceral and parietal endoderm in chimeras. Taken together, our data suggest that ES cell culture may represent trapped steady-state equilibrium between immediate early states of differentiation normally present in the early mammalian embryo. This state of equilibrium may exist in vivo for a limited period of time but in vitro is established by the active maintenance of blocks to differentiation in all available lineages and selective cell growth.

## Results

### Generation of a Sensitive Reporter of Early Endoderm Differentiation

**Figure 1:**
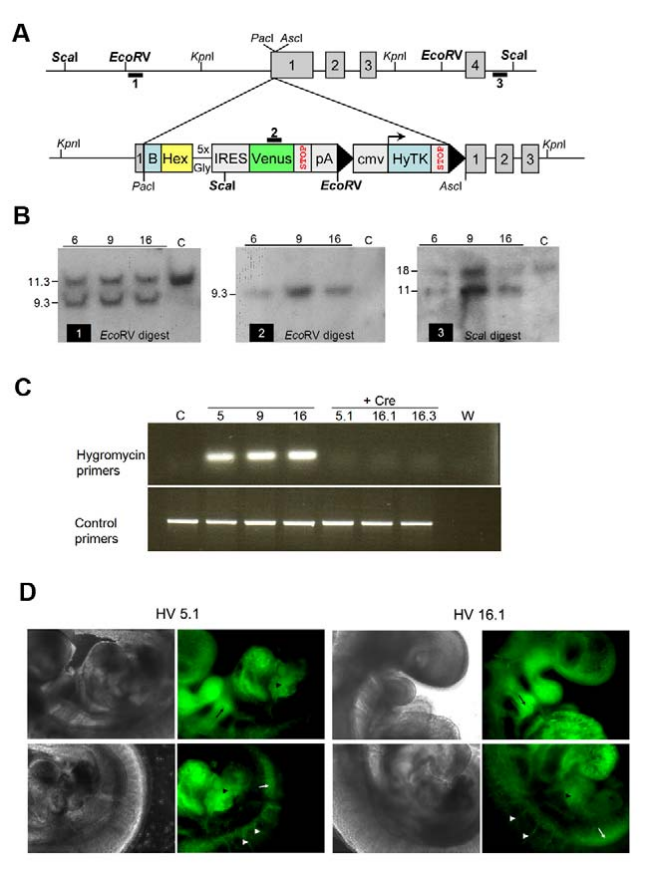
Targeting of the Hex locus with an amplified IRES Venus reporter. (A) Schematic representation of the gene targeting strategy. Hex cDNA tagged with a recognition site for the bacterial BirA ligase (B), followed by an artificial IRES sequence composed of a tandem array of reiterated 9 bp elements from the Gtx promoter and DNA encoding the fluorescent reporter, Venus, was inserted into the first exon of the *Hex* locus. (B) Southern blot analysis of targeted cell lines. Each blot depicted with an indication of the specific probe and digest. Genomic DNA digested with *EcoR*V was hybridised with either probe 1 to reveal WT (11.3 kb) or targeted (9.3 kb) bands, or probe 2 to produce a 9.3 kb band representing a single integration only in the *Hex* locus. Genomic DNA was also digested with *Sca*I and hybridised with probe 3 to reveal WT (17.8 kb) or targeted bands (11.5 kb). Genomic DNA from wild-type E14 cells is in the lanes labelled with a C. (C) Removal of selection cassette by transfection with the Cre recombinase. Following removal of the selection cassette through identification of Ganc^R^ clones a PCR based strategy was used to confirm excision. Primers specific for the hygromycin resistance gene were used alongside control primers to sites in the *Hex* promoter region. (D) HV reporter is faithful to Hex expression in chimeras. ES cells from two HV clones (5.1 and 16.1) were used to generate chimeras by morula aggregation. Embryos were obtained at E9.5 and imaged with fluorescence microscopy. Images show expression of Venus derived from two different clones in the thyroid (black arrow), intersomitic vessels (white arrowheads), the dorsal aorta region (white arrow), and liver primordium (black arrowhead).

To generate a reporter cell line that gives real time read outs of low-level early endodermal gene expression, we introduced a synthetic internal ribosomal entry site (IRES) designed to amplify translation upstream of a fluorescent reporter [31] into the first exon of the Hex genomic locus (Figure 1A). This IRES consisted of 10 tandem reiterations of nine base pair elements from the Gtx locus, previously shown to generate synergistic translation of a bicistronic message [32], driving expression of the enhanced fluorescent protein Venus. The reporter and a LoxP flanked selection cassette was inserted downstream of a tagged Hex cDNA to generate the Hex-IRES-Venus (HV) (Fig. 1A) targeting vector. The tagged Hex cDNA ensured wild-type levels of Hex expression and contains a sequence for in vivo biotinylation by the BirA ligase. ES cells were targeted and hygromycin resistant clones screened by Southern blot. Three clones were expanded for removal of the selection cassette by transfection with a plasmid expressing the Cre recombinase (Figure 1B, 1C). We confirmed that all three clones had a normal karyotype and contained the modification based on direct sequencing of the region containing the insertion (Figure S1 and unpublished data).

To confirm that the expression of the Venus allele reflects endogenous Hex expression [28],[33],[34], we used two HV clones to generate chimeras and examined the sites of high-level Venus expression during embryonic development. As expected, Venus expression was detected in the pharyngeal pouch endoderm, endocardium, inter-somitic vessels, and dorsal aorta (Figure 1D). We also tested the expression of the Venus allele during differentiation of the HV cells towards ES cell derived ADE that normally expresses high levels of Hex. This protocol was established with another Hex reporter line, Hex RedStar (HexRS), and requires 5 d of continuous exposure to the Nodal related TGF-β, activin [30]. Thus we differentiated these cell lines alongside HexRS reporter cells and examined the activin dependence of Venus expression (Figure S2). We also confirmed that this high level of Venus expression reflected quantitative induction of both endogenous Hex and another anterior endoderm marker Cerberus (Figure S2). Interestingly, while high levels of fluorescence and the expression of Hex and Cerberus mRNA required activin, low levels of Venus fluorescence were detected in the absence of activin. The detection of this level of Venus expression in the presence of low levels of Hex mRNA suggests that this reporter is indeed extremely sensitive to the low levels of Hex transcript produced in the absence of activin, earlier in differentiation, and in undifferentiated ES cells.

### Low Levels of Hex Expression Define a Unique Sub-population of Undifferentiated ES Cells

**Figure 2:**
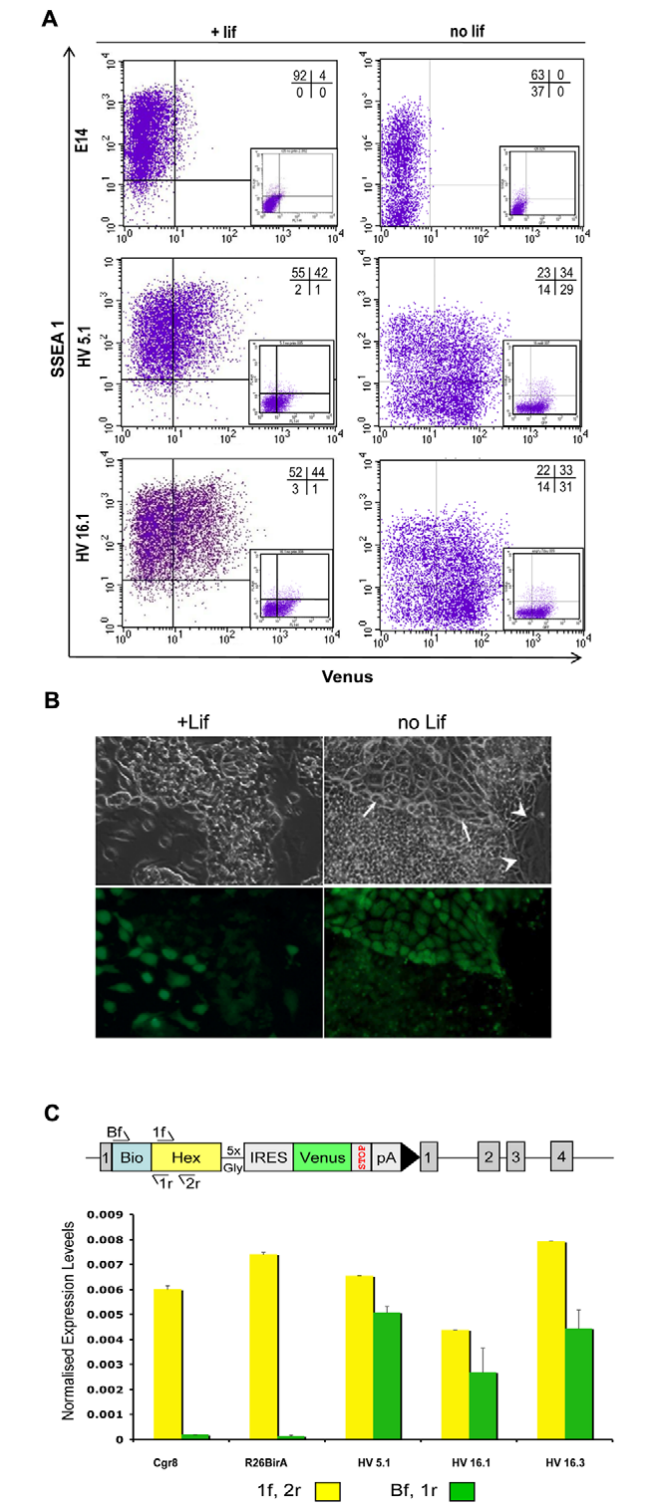
Expression of Venus in a subpopulation of SSEA 1 positive HV cells under self-renewing conditions. (A) Flow cytometry of two independent HV clones (HV 5.1 and HV 16.1) cultured either under self-renewing conditions or in the absence of LIF show the presence of a subpopulation of cells positive for Venus and/or the ES cell surface marker SSEA-1. Gates for expression of Venus and the presence of SSEA 1 were based on unstained E14 ES cells. Upon the removal of LIF for 3 d, the percentage of cells negative for SSEA 1 increased in both HV clones and the E14 cell line. (B) Fluorescence microscopy of the HV cell line in the presence or absence of LIF. Cultures were differentiated as (A). Note the brighter intensity of Venus in the tightly apposed pavement-like cells in the LIF negative culture (white arrows). Venus expression is absent from giant flat cells (white arrowheads). (C) Expression of the Venus transgene is similar to the low-level expression of the Hex cDNA. RNA was prepared from self-renewing cultures of three HV clones, parental R26BirA cells, and Cgr8 cells. Quantitative PCR analysis was carried out to monitor levels of mRNA derived from both targeted and untargeted alleles of Hex (1f, 2r) or targeted allele only (Bf, 1r). The schematic diagram depicts the different primers used. Values for each primer set used were normalised to the levels of Actin value obtained for each sample.

The low levels of Hex transcript observed in undifferentiated ES cells (Figure S2C) were sufficient to generate a significant Venus positive (V^+^) sub-population in undifferentiated ES cell cultures grown under standard feeder free conditions. Intriguingly, this population also expresses the ES cell marker, SSEA-1 (Figure 2A). Figure 2A shows that in the presence of the cytokine LIF, the majority of Venus-positive cells (70%) were also SSEA-1 positive (V^+^S^+^), while LIF withdrawal both increased the percentage of the population expressing high levels of the Venus transgene (mean level of fluorescence increases approximately 2-fold, Figure 2A) and led to a substantial increase in a second Venus positive population that is SSEA-1 negative (V^+^S^−^). Morphologically the majority of V^+^ cells grown in the presence of LIF appear indistinguishable from their V^−^ counterparts and the level of fluorescence in these morphologically normal V^+^ cells is substantially lower than that observed in cells that either appear differentiated or have been differentiated in response to LIF withdrawal (Figure 2B). Thus while the majority of the V^+^ population existing in ES cell cultures are indistinguishable from undifferentiated ES cells, we also observe differentiated cells expressing high levels of the Venus transgene (arrows in Figure 2B) that resemble the high-level Venus expressors generated in response to differentiation and that probably represent spontaneous PrEn differentiation.

As we were initially surprised by these observations, we asked whether the expression level of Venus RNA was equivalent to that generated by endogenous Hex. Using quantitative PCR, we compared the levels of Hex transcript from the wild-type and transgenic alleles to those from the transgenic allele only. We found that the endogenous Hex was expressed at extremely low levels with the transgene representing between 50% and 75% of this value (Figure 2C). Thus, Hex reporter gene expression appears to faithfully reflect the very low level of endogenous Hex transcript.

### The Venus Positive Population Represents an Early PrEn-Like State

**Figure 3:**
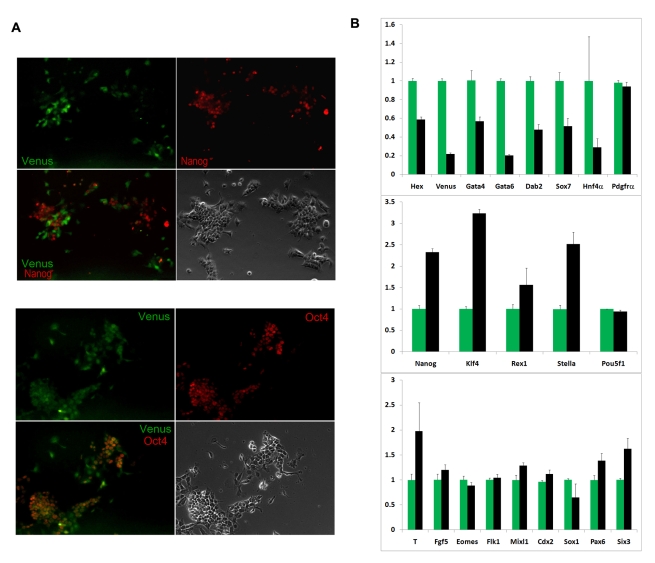
Venus positive population may represent an early state in PrEn differentiation. (A) Venus positive cells express Oct4, but not Nanog. Colonies of HV cells were fixed and immunostained for both Oct4 and Nanog. Primary antibodies specific to Oct4 and Nanog were detected using Alexa 568 conjugated secondary antibodies (red). Images include Venus fluorescence, antibody staining, overlay of Venus and antibody, and bright field for each cell line and the indicated antibodies. (B) Quantitative RT-PCR showing the relative expression of endodermal and pluripotency genes between Venus positive and negative cells obtained from the SSEA-1 positive fractions of two HV clones following flow cytometry. Quantitative PCR analysis was performed to compare transcript levels of Venus with PrEn (*Hex*, *Gata4*, *Gata6*, *Dab2*, *Sox7*, *Hnf4α*, and *Pdgfrα*), pluripotency (*Nanog*, *Klf4*, *Rex1*, *Stella*, and *Pou5f1*) and other lineage (*T*, *Fgf5*, *Eomes*, *Flk1*, *Mixl1*, *Cdx2*, *Sox1*, *Pax6*, and *Six3*) markers in purified cell fractions. Venus positive fractions are represented as green bars and Venus negative black bars. Transcript levels were normalised to the TBP value obtained for each sample. Normalised values are related to the level obtained for the Venus positive fraction in each case.

Since V^+^ cells were found abundantly in the SSEA-1 positive population, we asked whether this population expressed other markers of the undifferentiated state. Antibody staining for Nanog and Oct4, imaged alongside YFP/Venus fluorescence, indicated that while the Venus positive cells were also Oct4 positive, they expressed low levels of Nanog (Figure 3A).

To further address what the co-expression of these markers represented, we purified populations of cells from ES cell culture based on the expression of the Venus transgene and SSEA-1 by flow cytometry. Quantitative real time PCR based on RNA extracted from both SSEA-1 positive fractions revealed that while Oct4 levels remained constant, the Venus positive fractions from two different clones expressed higher levels of the PrEn markers Gata4, 6, Dab2, Sox7, and Hnf4α and lower levels of ICM markers such as Nanog, Klf4, Stella, and Rex1 (Figure 3B). Interestingly, we observed no enrichment of epiblast, neural, or mesodermal markers in the V^+^S^+^ fraction (Figure 3B, bottom panel) indicating that this fraction likely contained only progenitor cells specific to PrEn differentiation. During pre-implantation development Gata6 expression precedes Pdgfrα in putative PrEn precursors[35] and our V^+^S^+^ and V^−^S^+^ fractions expressed the same low to non-existent level of this transcript supporting the notion that V^+^S^+^ fractions contains early PrEn progenitors. Interestingly we observed approximately a 2-fold change in Nanog transcript levels between the two populations, and thus while the V^+^S^+^ cells appear Nanog negative based on antibody staining, they still express some Nanog transcript.

**Figure 4:**
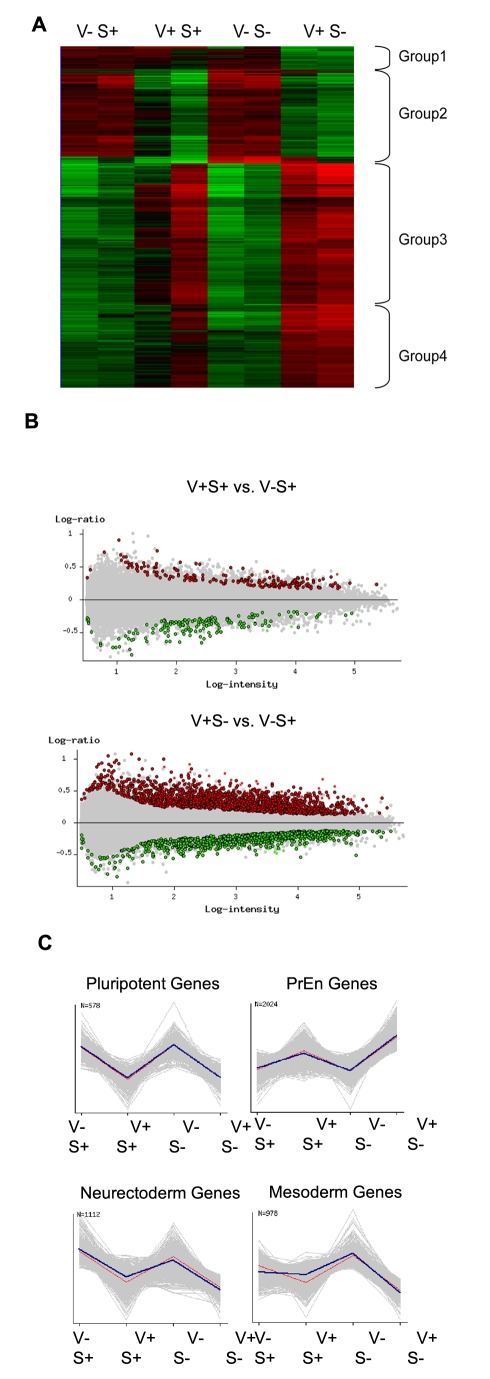
Microarray analyses of purified HV fractions. Analyses of global gene expression in fractions defined by expression of the Venus transgene and SSEA-1. HV ES cells grown under self-renewing conditions were fractionated by flow cytometry into four fractions based on Venus (V) and SSEA-1 (S) expression. RNA was isolated from the following fractions: V^−^,S^+^; V^+^,S^+^; V^−^,S^−^; V^+^,S^−^ and hybridised to a NIA Mouse 44K Microarray v2.1. (A) Heat map illustrating hierarchical clustering of differentially expressed genes identified in a pair-wise analysis of all four fractions. Significant changes in the expression of 2,169 genes (FDR <0.05) resulted in the identification of three to four expression groups, depending on whether clonal variation is taken into account. (B) Pair-wise comparisons (FDR <0.05, >1.5-fold expression levels) of the two ES cell populations, V^+^S^+^ and V^−^S^+^ depicted alongside the comparison between differentiated PrEn V^+^S^−^ fraction and the Venus negative ES cell fraction (V^−^S^+^). (C) Gene expression changes characteristic of PrEn, ICM/pluripotency, neurectoderm, and mesoderm genes (expression of individual markers are included as supplementary, Figure S3). Plots are shown comparing mean log intensity values of genes among the four populations. Error bars (see supplementary data) represent standard deviation between expression levels in independent clonal lines of HV cells.

To test the notion that this low level of transcription at the Hex locus producing the V^+^S^+^ fraction in ES cell culture represented an immediate early state in PrEn differentiation, we examined global differences in gene expression. RNA was isolated from all four fractions (V^−^S^+^; V^+^S^+^; V^−^S^−^; V^+^S^−^) in two independent clones of HV ES cells and hybridised to NIA Mouse 44K Microarray chips v2.3 (GEO Accession GSE13472) [36]. Hierarchical clustering of differentially expressed genes identified in a pair-wise analysis of all four fractions in both clones is shown in Figure 4A. Significant changes in the expression of 2,169 genes (FDR <0.05) resulted in the identification of three to four expression groups, depending on whether clonal variation is taken into account (Table S1). The greatest changes in gene expression were seen when the V^−^S^−^ and V^+^S^−^ fractions were compared (Figure 4B) with over a thousand genes changing in each direction. However, the differences between the two SSEA-1 positive fractions were relatively small, with only 139 non-redundant genes overexpressed and 123 underexpressed (FDR <0.05, 1.5-fold). While this group of genes is not large, what became apparent from inspection of the heat map in Figure 4A is that the majority of genes upregulated in the V^+^S^−^ cells are also marginally upregulated when the V^−^S^+^ to V^+^S^+^ fractions are compared. The size of this gene set varies somewhat depending on the particular clone, but this trend is particularly obvious when one considers sets of PrEn markers (Figure 4C and Figure S3). Thus for every PrEn marker examined we found subtle increases in gene expression were detected when the V^−^S^+^ and V^+^S^+^ fractions were compared and that these then translated into more robust increases in the V^+^S^−^ fraction.

We analyzed overrepresentation of Gene Ontology (GO) terms in the non-redundant genes that were overexpressed in the V^+^S^−^ and V^+^S^+^ fractions based on 1.5-fold change with a 0.05 FDR (Tables S2 and S3). We found that the V^+^S^+^ population expressed sets of genes that fell into major functional categories that were associated with “Cell adhesion” and “Cell migration.” The V^+^S^−^ fraction also featured these categories in addition to “Proliferation,” “Apoptosis,” and “Cytoskeleton.”

An equally consistent pattern of gene expression is observed in the set of ICM markers (contained within Group 2 in Figure 4A, Figure 4C, and Figure S3). Most of these genes were significantly down-regulated in both V^+^ fractions and remain high in the V^−^S^−^ fraction, indicating that this fraction contained a significant proportion of undifferentiated ES cells. This is consistent with the small number of gene expression changes (40 genes), with no significant pattern or common G0 annotation, that fluctuate with SSEA-1 when these two populations are compared to each other (Figure S4). While the majority of pluripotency genes were down-regulated in both V^+^ populations, there were some exceptions, including Oct4 and a class of differentiation inhibitors normally regulated by BMP4 including Id1, Id2, and Id3 [37]. Oct4 was expressed through the V^−^S^+^, V^+^S^+^, V^−^S^−^ fractions and down-regulated in V^+^S^−^, while the Id transcripts appeared to follow the PrEn genes, suggesting that they function to block neural differentiation in an early endoderm sub-population.

To confirm that early differentiation pattern exhibited in the V^+^S^+^ fraction was indeed an early state in PrEn differentiation, rather than a metastable pro-differentiation state similar to that described for the Oct4 positive populations that do not express Nanog, Rex1, or Stella [8]–[11], we examined the behaviour of gene sets representing other lineages in our data set (Figure 3C and Figure S3). Neither neuroectoderm nor mesodermal genes were upregulated in V^+^S^+^ fraction.

### Nanog Expression Suppresses the Venus Positive Early PrEn State

**Figure 5:**
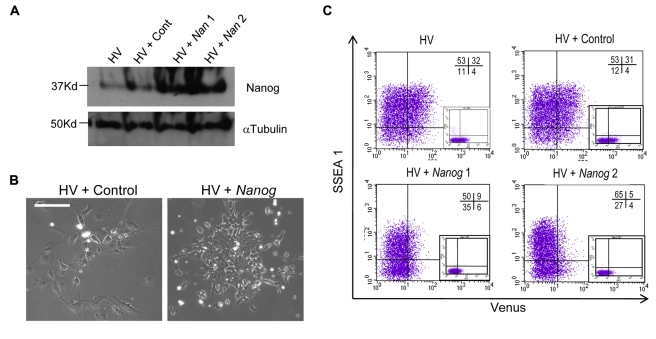
Nanog expression suppresses the Venus positive early PrEn precursor state. (A) Western blot demonstrating Nanog overexpression from the CAG promoter in two clones of HV cells. Control clones were derived in parallel with an empty vector. (B) Nanog overexpression makes HV ES cells resistant to LIF withdrawal. Nanog overexpressing and control cell lines were cultured in the absence of LIF for 10 d and assessed for ES cell-like morphology. (C) Nanog overexpression suppresses the V^+^S^+^ population. Expression of Venus and SSEA-1 were quantitated by flow cytometry in two independent clonal lines and compared to both control and parental cells.

As Nanog is rarely expressed in the Venus positive cells, we asked whether enforced Nanog expression would suppress baseline transcription at the Hex locus and thereby reduce expression of the Venus reporter. Nanog was misexpressed in HV ES cells under control of the CAG promoter driving an IRES puro cassette [38]. Western blotting showed increased levels of Nanog in 2 clones compared to parental and control cells (Figure 5A). As overexpression of Nanog in ES cells supports LIF independent growth [6],[38], we confirmed Nanog overexpression in the HV line by observing the persistence of ES cells following 10 d culture in the absence of LIF (Figure 5B).

Nanog overexpressing HV cells were grown in the presence of LIF and the fraction of these cultures that expressed the amplified Venus transgene quantitated by flow cytometry. In two independent clones we observed a dramatic reduction in V^+^S^+^ population (3–6-fold, Figure 5C), suggesting that Nanog can regulate low transcription at the Hex locus.

### Manipulation of FGF Signalling Alters the Levels of Venus Expression

**Figure 6:**
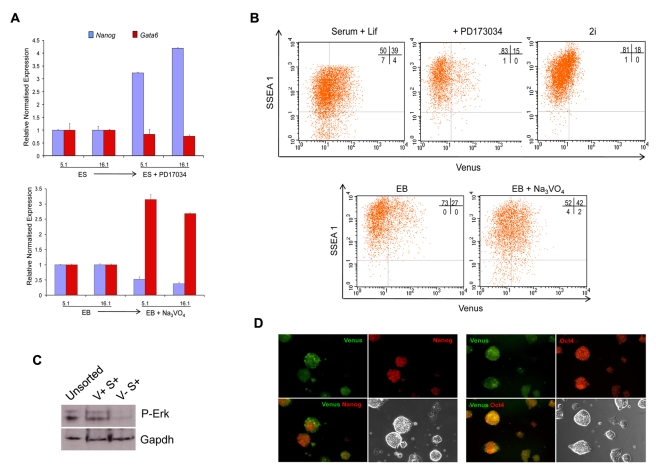
Manipulation of FGF signalling alters the levels of Venus expression. (A) FGF signalling modulates Nanog and Gata6 expression in HV cells. Inhibition of FGF signalling with PD173074 (10 nM) increases the levels of Nanog gene expression in two HV clones while slightly reducing low-level Gata6 expression. Conversely, potentiation of this pathway with the phosphatase inhibitor Sodium Vanadate (50 µM) in aggregate cultures (EB + Na_3_VO_4_) reduces the levels of Nanog while increasing those of Gata6. Transcript levels were assessed by qPCR and normalised to the TBP value obtained for each sample. Normalised values are related to the untreated sample for each clone. (B) The V^+^S^+^ fraction responds to FGF signalling. Cells grown as in (A) were subject to flow cytometry. Inhibition of the FGF pathway by PD173074 or culture in 2i reduces the extent of Venus expression, while Sodium Vanadate stimulates it. (C) Measurement of Phospho-Erk levels in V^+^S^+^ and V^−^S^+^ fractions shows an enrichment of activated Erk with Venus positive ES cells. (D) Venus cells persist in 2i culture. Immunocytochemistry of HV cells in 2i culture show the persistence of some Venus cells that have lower levels of Nanog expression, whereas Venus and Oct4 co-express.

The ability of Nanog to suppress early Hex positive endoderm states is consistent with both the mutually exclusive nature of Nanog and Gata6 expression in vivo [17] and the ability of Nanog to suppress Gata6 positive PrEn differentiation, in vitro [39]. The shift between a Nanog positive ICM-like state and Gata6 positive PrEn is also regulated through FGF signalling via the Grb2/Mek pathway [17],[40]. As the V^+^S^+^ population appeared to be an immediate early state of PrEn differentiation in which extremely low levels of PrEn determinants (e.g. Hex) are expressed, we wanted to ask whether FGF signalling promoted this state or acted to push cells already in this state further into differentiation. Thus we examined whether FGF signalling could alter the dynamics between the V^+^ and V^−^ states within the S^+^ population by culturing HV cells in the presence of the FGFR inhibitor PD173074 [41] for 48 h. As expected, treatment of HV cultures with PD173034 suppresses background levels of PrEn differentiation at the level of Gata6 and Nanog transcription (Figure 6A). However, the inclusion of PD173034 in these cultures also reduced the size of V^+^S^+^ fraction (Figure 6B). In addition to feeder free serum and LIF containing media, ES cells can be cultured in minimal serum free media (referred to as 2i) containing the MEK inhibitor PD0325901 that targets the phospho ERK branch of the FGF pathway and the GSK3-β antagonist CHIR99021 [42]. When maintained in 2i culture, cells are grown under constant blockade to phospho-Erk signalling. As expected the culture of HV cells under these conditions resulted in a significant reduction in the V^+^S^+^ population (Figure 6B). Thus induction of a robust V^+^S^+^ state of low-level PrEn transcription requires FGF signalling. However, while the expression of the Venus transgene is greatly reduced in 2i, it is still present (Figure 6B, 6D). Moreover, while antibody staining and microscopy of ES cell colonies grown in 2i showed uniform morphology, no detectable Gata6 expression and reduced Nanog heterogeneity, Venus positive cells were visible within these colonies and this Venus positive expression was rarely found within cells expressing high levels of Nanog (Figure 6D). While expression of the Nanog protein in the V^+^S^+^ fraction appears largely reduced or absent, we have been unable to detect differences between 2i generated V^+^S^+^ and V^−^S^+^ cells by RT-PCR (unpublished data). This is not surprising as the amplified transgene was already detecting very low transcript levels in serum and the levels of Venus expression in 2i were 2–3-fold lower.

We confirmed the ability of Fgf signalling to regulate the V^+^S^+^ population by treating suspension cultures with the phosphatase inhibitor sodium vanadate to stimulate the FGF/Grb2/Mek pathway. Treatment of cell aggregates with sodium vanadate in the presence of LIF has been shown to repress Nanog and stimulate PrEn differentiation [40]. Thus when HV cells were cultured under these conditions, the addition of sodium vanadate suppressed Nanog expression, lead to a significant increase in Gata6 (Figure 6B), and produced a 25% increase in the percentage of the culture that was V^+^S^+^ (Figure 6B). These observations appear specific for early PrEn, as treatment of Sox1-GFP cells with either PD173034 or sodium vanadate had little effect on GFP expression (unpublished data). Taken together these data support the notion that low-level transcription at PrEn promoters such as Hex is dependent on signalling via the FGF/Grb2/Mek pathway. Interestingly when ES cells were fractionated based on the Venus transgene, the V^+^S^+^ cells contained almost all detectable phospho-Erk activity (Figure 6C).

### Reversibility of Early PrEn States in vitro

**Figure 7:**
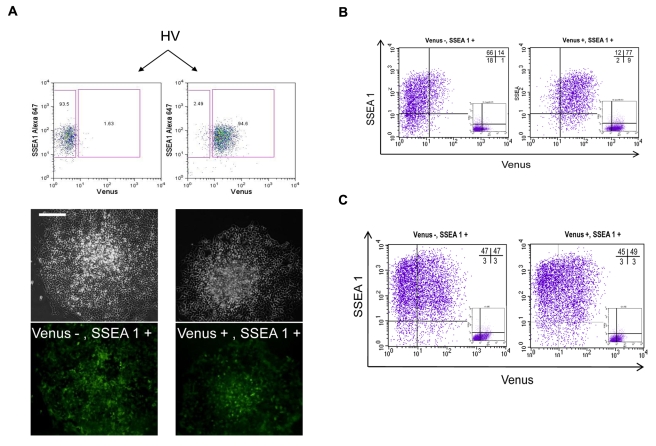
Reversibility of Venus positive and negative populations. (A) Reconstitution of Venus distribution from single V^+^S^+^ or V^+^S^−^ cells. HV cells cultured under self-renewing conditions were subjected to flow cytometry to separate Venus positive and negative subpopulations within the SSEA-1 positive fraction. A sample purity check is shown in the top panel. Representative clones produced from each fraction plated at clonal density and imaged by fluorescence microscopy are shown. (B) Flow cytometry on cells from each fraction 24 h after plating. (C) Flow cytometry on cells plated at single cell density in 96 well plates from each sorted fraction. Cells were cultured for 10 d following plating and 12 wells derived from each fraction were subjected to flow cytometry. All appeared identical and a representative image of each is shown in the figure.

Heterogeneous ES cell states have been observed with respect to Nanog, and while the Nanog expression state appears reversible, there are significant differences in the ability of Nanog positive and negative cells to clonally reconstitute each other in vitro [8]. Thus we asked whether the V^+^S^+^ population and V^−^S^+^ could efficiently interconvert. To test this we plated cells sorted by flow cytometry clonally and assessed the extent to which colonies could re-establish steady-state equilibrium. While the plating efficiency of the V^+^S^+^ fraction was reduced and produced 4-fold less colonies than the V^−^S^+^ fraction, both fractions gave rise to identical colonies that contain equivalent populations of V^+^ and V^−^ cells (Figure 7A, Table 1). Thus, while there appears a difference in the colony forming potential of the two fractions, once colony formation is initiated, the two cell types are identical in their ability to give rise to each other.

**Table 1:** Numbers of clones produced from clonal density plating of cell from FACS purified fractions of the HIV cell line.

	Number of Clones Obtained	% of Clones Fluorescent by Microscopy
Venus−, SSEA1+	90	100
Venus+, SSEA+	21	100

To determine the length of time required for the two states to interconvert we purified populations V^+^S^+^ and V^−^S^+^ cells and examined the extent to which the original distribution was re-established and observed significant changes in both populations within 24 h of plating (Figure 7B). To further test the notion that V^+^ and V^−^ cells were both equally capable of clonally regenerating the equilibrium normally present in ES cell cultures, we deposited single cells in 96 well plates following sorting by flow cytometry. Consistent with our previous observations, single V^+^S^+^ and V^−^S^+^ cells were equivalent in their ability to regenerate normal Venus distribution upon expansion in 30 independent clonal cultures (Figure 7C). In this instance we did not detect a plating difference in the populations and approximately 16% of the deposited cells survived to give rise to day 10 cultures (unpublished data). Taken together these data support the notion that the V^+^S^+^ fraction represents an early state of PrEn differentiation that exists in equilibrium with other cell states present in ES cell cultures.

### Early PrEn States Exhibit Functional Bias

The ability of these populations to interconvert in vitro combined with their subtle differences in gene expression lead us to ask if there was any functional significance to this low level of PrEn gene expression. As ES cells are defined based on their ability to contribute to all tissues of the future conceptus in chimeras, we asked whether the embryo contribution activity of ES cells was contained in either V^+^S^+^ or V^−^S^+^ fraction or both. Initially we injected purified fractions of HV ES cells into Rosa26 blastocysts that constitutively express β-galactosidase (β-gal) and examined embryos at 9.5 *dpc* for ES cell (β-gal negative) contribution (Table S4 and Figure S5). In these experiments the Venus positive fraction never gave rise to high-contribution chimeras and less than half of the injected embryos showed any contribution whatsoever. This contrasted starkly with the Venus negative fraction, which contained cells that were effective at generating high-contribution chimeras. Thus the modest changes in gene expression that accompany basal level PrEn expression interfere with the capacity of these cells to actively contribute to blastocysts.

**Figure 8:**
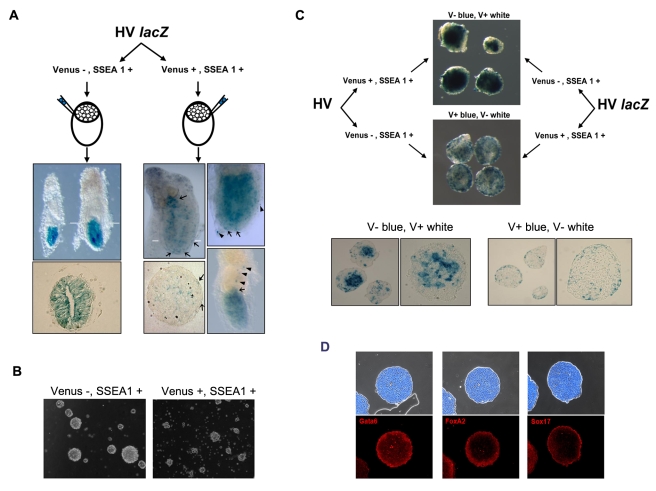
Functional differences between purified Venus positive and negative ES cells. (A) V^+^S^+^ and V^−^S^+^ ES cells contribute differently to embryos in morula aggregation. HV cell lines constitutively expressing β-Geo from the CAG promoter (HV lacZ) were fractionated into V^+^S^+^ and V^−^S^+^ and their ability to contribute to chimeric embryos assayed by morula aggregation. Within an hour of separation by flow cytometry, cells from each fraction were aggregated with wild-type F1 morulae. Following transfer into pseudo-pregnant mice, resultant embryos were harvested at E6.5 and subjected to X-gal staining. Representative embryos derived from each population are shown. White bars indicate the plane of section shown in the panel beneath specific embryos. Black arrows show the presence of LacZ positive cells in the visceral endoderm. Black arrowheads show the presence of LacZ positive cells in the parietal endoderm. (B) Only V^−^S^+^ fraction forms normal spherical EBs. Fractionated HIV ES cells were cultured for 4 d as aggregates in the absence of LIF. (C) V^+^S^+^ cells contribute to the presumed visceral endoderm in chimeric EBs. When V^+^S^+^ cells were recombined with V^−^S^+^ cells immediately following sorting, they formed normal EBs and the V^+^S^+^ cells move preferentially to the outside to form the presumptive visceral endoderm. The V^−^S^+^ fraction of HVlacZ cells was combined with an equivalent number of V^+^S^+^ HV cells (top) or V^+^S^+^ HVlacZ cells recombined with V^−^S^+^ HV (bottom). EBs were stained with X-gal and representative sets shown. The bottom panel shows sections through representative chimeric EBs. (D) Sections of EBs grown under the same conditions as in part (C), showing that the outer layer consists of visceral endoderm as marked by *Gata6*, *FoxA2*, and *Sox17* immunostaining (shown as red). Bright field/DAPI composites of each section are shown above.

The loss in ability to contribute to blastocysts generated in this transient PrEn-like state was interesting, but we wanted to establish if these cells had gained new properties. To ascertain this we generated cell lines that both contained the HV cassette and constitutively expressed β-gal as a lineage label. We used this cell line for morula aggregation and obtained the chimeric embryos shown in Figure 8A. These results validate our observations obtained with blastocyst injection and indicate that the V^−^S^+^ fraction is particularly effective at contributing to the epiblast (Table 2).

**Table 2:** Assessment of lineage contribution of V^+^S^+^ and V^−^S^+^ cells from the HV LacZ line at 6.5 dpc.

	Venus+, SSEA+	Venus-, SSEA1+
*n*	120	69
No contribution	46%	25%
Low-medium	47%	0%
Medium-high	7%	75%
VE/PE contribution	10%	0%

Table shows the numbers of resultant embryos from aggregation with wild-type morulae following fractionation based on Venus and SSEA1 expression by flow cytometry. Percentages of embryos with LacZ positive cells detected in the Visceral or Parietal (VE/PE) endoderm are shown.

Interestingly, while the V^+^S^+^ cells did not effectively contribute to the epiblast, V^+^S^+^ ES cells were found in both the visceral and parietal endoderm (Figure 8A, Table 2), suggesting that their reduced ability to contribute to the epiblast may reflect a change in potency. To confirm this observation by another method we asked about the potency of these fractions to differentiate in EB aggregates. However, while V^−^S^+^ cells generated normal EBs, the V^+^S^+^ cells formed small irregular aggregates (Figure 8B), suggesting that the adhesive properties of the cells within these fractions were different. This would not be surprising as early PrEn delaminates from the ICM during the transition between ICM and epiblast and this cell sorting behaviour is reproduced in EB culture where the VE is always found on the outside. Thus when Xen (extra-embryonic endoderm) cells are mixed with ES cells, the Xen cells segregate to end up on the outside layer [43] of the EB. In a similar way, we used HV lacZ ES cells to ask whether the V^+^S^+^ fraction would preferentially segregate to the outside of chimeric EBs. Figure 8C shows that this is indeed the case. Labelled fractions of V^+^S^+^ cells ended up on the outside of chimeric EBs, while the reciprocal fraction of V^−^S^+^ populated the centre of the aggregate. We then stained these EBs with three antibodies to the endoderm markers Gata6, FoxA2, and Sox17 to confirm that these outside cells were endoderm and indeed all three markers were expressed throughout the outside layer (Figure 8D). Taken together our data support the notion that the reversible and immediate early PrEn state marked by low-level transcription at the Hex locus is biased towards the formation of extra-embryonic endoderm.

## Discussion

In this paper we have used translational amplification to detect an immediate early and reversible state in PrEn differentiation that appears an inherent component of standard ES cell culture. The existence of ES cell precursors to this lineage is supported by the observed heterogeneous expression of other PrEn genes, Lefty1, Cerl, and Gata6 in the ICM of blastocyst stage embryos, the stage from which ES cells are derived [17],[44]–[46]. Cells in this ES cell state express low levels of PrEn markers such as Hex and maintain expression of some standard ES cell markers such as Oct4 and SSEA-1. These cells can be isolated based on the expression of an amplified Hex Venus transgene and SSEA-1 (V^+^S^+^) and exist under ES cell conditions in a steady-state equilibrium with at least one other more ICM-like cell state, V^−^S^+^. When purified V^+^S^+^ or V^−^S^+^ cells are placed back into self-renewing conditions, individual cells from purified fractions of either cell type regenerate their counterparts. However, when these fractions are placed into differentiation either in vivo or in vitro, the V^+^S^+^ population tends to colonize the PrEn lineages, while V^−^S^+^ cells tend towards epiblast.

**Figure 9:**
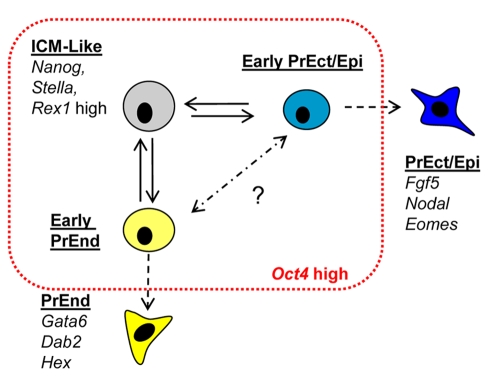
A model for the dynamic equilibrium that exists in ES cell culture. The schematic diagram depicts the potential cell subtypes that make up ES cell culture. The red line represents the boundary established by the culture conditions. We depict an early PrEn precursor cell defined by the V^+^S^+^ phenotype in light yellow, expressing low levels of PrEn determinants such as Hex and Gata6. This cell type is shown in equilibrium with an ICM-like cell. A hypothetical PrEc cell implied by the findings of others is indicated in blue.

A number of recent studies have suggested that ES cell cultures are heterogeneous and can be split into two developmental states, one that resembles the ICM and the other early epiblast or PrEc. Thus it has been suggested that ES cell cultures can be split based on Rex1 and Oct4 [9], into Rex1, Oct4 positive ICM, and Rex1 negative Oct4 positive PrEc. Similar observations have also been made with an ICM-specific, Stella-GFP reporter [10] that can be used to split ES cell cultures into Stella positive ICM-like and Stella-negative epiblast-like. In both instances, the ICM state appears to express higher levels of Nanog and this observation is consistent with the heterogeneous expression of Nanog reporter ES cells [8],[12]. Elevated levels of Nanog are also associated with a reduced probability of differentiation leading to the suggestion that ES cells exist in equilibrium between a stable self-renewing, ICM-like state referred to as the “ground state” and a transient metastable intermediate that is both able to revert to the self-renewing state or proceed into differentiation [8],[11],[47]. The transition between the ground state and this metastable pro-differentiation intermediate is thought to be regulated by FGF/Erk signalling [47],[48]. While our data do not provide insight into the dynamics of the entire Nanog low population, it suggests that a sub-fraction of low Nanog cells represents PrEn precursors, in addition to the already characterized PrEc precursor population. Moreover, in PrEn precursors, the Nanog low population can itself be split based on the expression of Oct4 or SSEA-1 into a state expressing reasonably high level of PrEn genes (V^+^S^−^), and a less differentiated cell type exhibiting a PrEn bias, but with similar regenerative capacities to the Nanog high population (V^+^S^+^). We believe that a similar early precursor may exist to the PrEc lineage (Figure 9), and while we have no direct evidence for this, we did observe Oct4 positive cells that neither expressed Nanog nor the Venus transgene and there also appears a slight enrichment of early neural markers in the V^−^S^+^ population (Figure 4). However, we were not able to discern this state based on SSEA-1 expression, as a number of both ICM and PrEc markers are expressed at equivalent levels in the V^−^S^+^ and V^−^S^−^ fractions. Thus while SSEA-1 may be an effective marker for undifferentiated cells when used in combination with a PrEn marker, its utility may be limited to this lineage.

In addition to expressing slightly increased PrEn gene expression, V^+^S^+^ cells also contain almost all the phospho-ERK activity in our ES cell cultures (Figure 6). As this population does not express elevated levels of transcripts specific to other lineages, it suggests that FGF signalling does not promote the formation of a general metastable pro-differentiation state but rather supports the formation of the V^+^S^+^ reversible PrEn intermediate. How then do we explain the requirement for FGF/Erk signalling in ES cell differentiation towards other lineages [48],[49]? One possibility is that V^+^S^+^ cells produce additional factors required for these lineages.

The notion that a Nanog positive, ICM-like population of high probability self-renewing cells is a developmental ground state is supported by the expansion of this state in the presence of a blockade on the major signalling pathways known to promote ES cell differentiation, the MAP kinase/ERK cascade and GSK3β [42],[50]. Thus when extrinsic inputs are reduced, ES cells revert homogenously to this Nanog positive ground state. Interestingly, while these 2i conditions reduced the extent of the Venus positive population in steady-state culture, it remains a significant component of ES cell culture and exclusive of high Nanog expression. We also observed that single cells from either the V^+^S^+^ or V^−^S^+^ fractions were both equally effective at generating clonal cultures with the normal range of Venus expression and in no cases did V^+^S^+^ cells give rise to differentiated colonies. As a result we conclude that both fractions are equivalent with respect to their capacity for ES cell self-renewal and V^+^S^+^ cells do not constitute a metastable early state in differentiation but rather an integral uncommitted component of ES cell culture. In the model shown in Figure 9, we suggest that a similar uncommitted and self-renewing state may exist in the direction of ectodermal differentiation and we imagine the ground state could consist of at least three distinct populations in equilibrium. These cell states would all appear as morphologically undifferentiated and express equivalent levels of Oct4.

Based on the equivalent regenerative capacity of V^+^S^+^ and V^−^S^+^ cells, the small number of significant gene expression changes, and the identical morphology, we assume that these two cell states have not drifted significantly apart. Rather these states may represent distinct reversible transcriptional signatures affecting key lineage regulators. Comparison of the differences in gene expression between the V^+^S^+^ and V^−^S^+^ fractions supports this idea. Every PrEn marker present in our data set increased in the differentiated V^+^S^−^ cells and importantly showed small but consistent increases when the V^+^S^+^ fraction was compared to V^−^S^+^ cells. As a result we believe that ES cells in culture consist of a mixture of early self-renewing precursors that can alternatively express low-level transcription of different lineage-specific promoters related to the states surrounding the early blastocyst (Figure 9). Whether the ICM-like state is central to this equilibrium remains to be seen.

The model in Figure 9 represents a stable dynamic system in which the transcriptional state of individual cells shifts, but only within the boundaries defined in red. This suggests that the behaviour of transcriptional networks downstream of Nanog, FGF signalling, and other key ES cell regulators produce an attractor or attractor states occupied by these cell types. The existence of multiple sub-states within a single ES cell basin of attraction or multiple interrelated attractors representing distinct lineages could account for pluripotency. Similar dynamic models have been extensively discussed as a means to explain stem or progenitor cell potency (reviewed in [51],[52]). In these models, the capacity of a progenitor cell to differentiate into multiple lineages is determined by a form of “multi-lineage priming” [53], in which cells fluctuate through the early states of multiple lineage programs but remain within a stable basin of attraction. When the culture is removed from the constraints of self-renewal, lineage primed states drive commitment to a direction of differentiation based on the location of a cell in a specific state or attractor. In ES cells, early V^+^S^+^ PrEn would become extra-embryonic endoderm and early PrEc would become epiblast. However, when maintained in ES cell culture, cells transit between these states. One possible mechanism for the movement of cells from one state to another would be the combination of stochastic changes in low-level gene expression or noise, combined with positive feedback loops. Indeed this sort of model has been used to explain the existence of a stable attractor and associated lineage primed states in EML cells, a haematopoietic progenitor cell line [54], and as the basis for heterogeneity in Nanog expression in ES cells [11]. However, both these cases consider the ability of stochastic variation to drive the formation of a single stable attractor. While the small changes in lineage transcription observed in our data set would be consistent with a stochastic model, the ES cell model described in Figure 9 would require both cross-repression and additional positive feedback loops to drive these random changes in gene expression down multiple distinct routes. An alternative mechanism that might explain the ability of cells to transit between multiple states is oscillating gene expression. It was recently suggested that Hes1 expression can cycle in ES cell culture [13], although the link between this oscillation, low-level gene expression, and developmental bias is not clear. Regardless of whether the gene expression changes are deterministic or random, feedback between cell types may help to stabilize this heterogeneous culture system. The existence of a paracrine inter-dependent equilibrium would suggest that the culture conditions have selected for the stable coexistence of mutually dependent and metastable cell types that only transiently exist in vivo.

Our observation that the V^+^S^+^ fraction preferentially contributes to the VE when mixed with more ICM-like cells indicates that low-level lineage-specific changes in gene expression have functional consequences. That we have observed a direct contribution of ES cells to both visceral and parietal endoderm also has implications for canonical definitions of pluripotency. Pluripotency is defined based on the ability of ES cells to contribute to the embryonic but not extra embryonic lineages and our observations suggest this definition may need to be somewhat modified. Alternatively it might be more appropriate to consider ES cells as closer to totipotent, but that the pluripotent ICM fraction of ES cell cultures has a competitive advantage when tested in chimera generation. In support of this idea, Beddington and Robertson originally observed ES cell contribution to all the extra-embryonic lineages, but in particular to parietal endoderm [23]. However, these observations have been seen as the exception rather than the norm because of the low-level contribution observed. As the principle significant gene expression changes observed in the V^+^S^+^ fraction are related to adhesion and migration (Table S2), this might explain the decreased capacity of these cells to incorporate into a host ICM and instead colonize the extra-embryonic endoderm. The lower level of endodermal contribution we observe in chimeras suggests that even in the PrEn, V^+^S^+^ ES cells may be at a proliferative disadvantage.

The observation that some ES cells retain the capacity to contribute to the extra-embryonic lineages begins to resolve a number of conflicting observations. Why should ES cells be able to generate PrEn in vitro but not in vivo? Moreover, as it has recently been shown that VE can contribute to the embryonic gut [55], the distinction between visceral and definitive endoderm begins to blur and the inability of ES cells to contribute to the VE becomes more puzzling. Chazaud et al. observed that heterogeneous expression of Nanog and Gata6 in early blastocysts was dependent on Grb2-MAPK signalling and suggested that the reason that ES cells are unable to colonize the PrEn meant they had lost the capacity to respond to this signal [56]. Our observations reconcile these apparent discrepancies. ES cells exhibit the same heterogeneity as the early blastocyst and respond to the same signalling pathways. They have the capacity to contribute to both epiblast and PrEn lineages in vivo and in vitro, but when mixed populations of ES cells are combined with embryonic ICM in a situation where a limited number of cells can be accommodated, a competition ensues that is regulated by a combination of differential adhesion and proliferation. That we observe cell sorting in EB culture also provides direct evidence, albeit in vitro, for the differential adhesion model proposed for the resolution of early PrEn and PrEc in the mammalian blastocyst in this same paper [56]. That this occurs once cells enter differentiation, is consistent with a requirement for sustained FGF signalling for commitment and segregation of the PrEn lineage in cultured blastocysts [57].

The capacity of V^+^S^+^ cells to colonize the exterior of EBs and extra-embryonic endoderm in chimeras is similar to the properties of extra-embryonic endoderm (Xen) cells derived from the mammalian blastocyst [43]. Xen cells are more parietal than visceral in character, whereas our cells expressed more anterior visceral or early PrEn markers. However, we have not attempted to culture the more endodermal V^+^S^−^ cells and it will be interesting to see if these cells can be expanded in vitro. Whether they can retain their visceral or primitive qualities in absence of a more epiblast-like population remains to be seen. Interestingly when parietal endoderm is grafted next to epiblast, it becomes visceral and when VE is removed from epiblast it becomes parietal [58].

We recently performed a genome wide screen looking for Hex targets in ES cells and found a number of genes with ICM expression patterns [59], consistent with the notion that as Hex levels build up it would repress ICM identity and promote commitment to the PrEn lineage. As these targets appeared conserved in evolution, it would seem likely that they are not specific to ES cells and that the same low-level expression states might exist for a limited window of time in vivo. Recent time lapse studies of pre-implantation development suggest that cells that are initially Pdgfα PrEn can revert to ICM [35], indicating that at least some reversible sampling of these low-level transcription states might occur in vivo. Although Pdgfrα appears downstream of the fluorescent signal observed here, the dynamic nature of cell fate specification appears similar. In ES cells these events would have been amplified, as potential developmental intermediates have been trapped and are maintained in a stable dynamic equilibrium. In this way embryo-derived stem cell lines and ES cell differentiation may be providing access to potential “transition states,” required for lineage specification in vivo.

## Methods

### ES Cell Culture and Differentiation

ES cells were cultured on 0.1% gelatin-coated flasks or plates (IWAKI) in Glasgow modified Eagle's medium (Gibco) containing non-essential amino-acids, glutamine and sodium pyruvate, 0.1 mM mercaptoethanol, and 10% Fetal Calf Serum (FCS) together with LIF [30],[60]–[63].

ES cells were differentiated toward ADE in aggregation culture according to [30]. Differentiation towards PrEn in the presence of sodium vanadate is as described in [40]. LIF withdrawal in monolayer culture was done according to [25].

### Generation of Vectors and Cell Lines

The 5′ and 3′ arms used for homologous recombination were described by Martinez Barbera et al. [33] with *Asc*I and *Pac*I sites inserted downstream of the Hex ATG (a gift from Shankar Shrinivas). A Hex cDNA with a recognition sequence for bacterial BirA ligase was linked via an artificial IRES consisting of a tandem array of repeated Gtx sequences to the gene encoding Venus followed by a cytomegalovirus driven hygromycin-thymidine kinase dual selection cassette flanked by loxP sites. This entire cassette was fused in frame with the ATG of Hex in the targeting vector. Following electroporation into R26 BirA cells, a cell line that expresses bacterial BirA ligase from the ROSA26 locus, hygromycin resistant clones (200 µg/ml) were expanded for Southern analysis to identify correct targeting events. The selection cassette was then excised from two clones, HV 5 and HV 16, from which Gancyclovir resistant clones were selected for further analysis. HV cells overexpressing Nanog were generated by electroporation with a vector containing the Nanog cDNA under the control of a CAG promoter and upstream of IRES Puro cassette followed by selection in puromycin (2 µg/ml) for 2 wk. HV cells constitutively expressing the *LacZ* gene were generated by electroporation with a vector containing a CAG driven β-Geo cDNA followed by selection in G418 (150 µg/ml) for 2 wk.

### Immunocytochemistry and Flow Cytometry

Cells grown in 12 well plates were washed 2× in PBS before fixation in 4% paraformaldehyde. Cells were then permeabilised in PBST (1× PBS, 0.1% Triton X (Sigma)). Blocking was performed by adding 1% Bovine serum albumin (Sigma) in PBST solution to the fixed cells for 30 min at room temperature (rt). Primary antibodies were added at a dilution of 1∶1000, and incubation continued overnight (o/n) at 4°C. Following 3×10 min washes in PBST, Alexa568 conjugated secondary antibodies diluted (1∶1000) in blocking solution were added to the cells and incubation took place at rt for 1 h. Also included at this step was DAPI solution (1∶1000). Finally, cells were washed 3 times, then stored in PBS. Primary antibodies used were mouse anti-Oct3/4 (Santa Cruz) and rabbit anti-Nanog peptide specific antibodies (a gift from Ian Chambers) [8]. Secondary conjugated antibodies (Alexa568) against mouse and rabbit were obtained from Invitrogen.

ES cells or EBs were collected into Cell Dissociation Buffer (Gibco) and incubated at 37°C for 10 min. Single cells suspension was achieved by gentle repeated pipetting. Following washes in PBS, cells were resuspended in 500 µl FACs buffer (1× PBS, 10% FCS) and 7AAD solution (BD Pharmingen, 5 µl/1×10^6^ cells) to exclude dead cells. Analysis of fluorescence took place in a FACSCalibur flow cytometer (BD Biosciences). Dotplots were generated using CellQuest software (BD Biosciences).

In the case of additional labelling of specific cell surface proteins, primary antibodies were added at a dilution of 1∶1000 to cells resuspended in FACs buffer. Incubation took place for 10 min on ice. Following three washes in FACs buffer, cells were resuspended in fresh FACs buffer containing appropriate conjugated antibody at a dilution of 1∶1000 and incubated as before. After three washes in FACs buffer, cells were finally resuspended in 500 µl FACs buffer and analysed as above.

For collection of populations, cells were prepared as above and subjected to flow cytometry using the MoFlo MLS high speed sorting apparatus (DakoCytomation). Cells were collected in FACs buffer and stored on ice for further analysis.

### Chimera Generation

Chimera mouse generation was performed by morula aggregation with or injection of ES cells into host blastocysts. Injected or aggregated blastocysts were then transferred into pseudopregnant recipient mothers. Embryos were dissected at the stages indicated in the figures and imaged by fluorescent and conventional microscopy.

### X-gal Staining and Histology

X-gal staining of embryos and EBs was performed as follows. Embryos and EBs were washed in PBS solution (80 mM sodium phosphate, 15 mM potassium phosphate, 27 mM KCl, and 1.37 M NaCl), then fixed with X-gal fix solution (1× PBS, 2 mM MgCl2, 5 mM EGTA, 1% paraformaldehyde, 0.2% Glutaradehyde, 0.02% NP-40) at 4°C for 20 min. Following 3×20 min washes in PBS they were then stained with X-gal staining solution (5 mM potassium ferricyanide, 5 mM potassium ferrocyanide, 2 mM MgCI, 0.01% sodium deoxycholate, 0.02% Nonidet P-40 (NP-40) in PBS) o/n in the dark at rt. Following 3×5 min washes in PBS, stained embryos or EBs were then fixed in 4% paraformaldehyde.

X-gal stained, paraformaldehyde fixed embryos were embedded in paraffin wax and sectioned transversely in a microtome at 7 micron intervals. X-gal stained or unstained EB or embryos were also cryosectioned. Samples were sunk in 30% sucrose in PBS, frozen in Tissue Teck, and sections were cut on a Cryostat (Leica). Sections were collected on poly lysine microscope slides (VWR International), air-dried for 30 min to 1 h, and stored at −20°C until used. Immunocytochemistry was performed essentially as described above for cells.

### Microarray Analysis

RNA was extracted from different cell populations using Trizol™ (Invitrogen) and precipitated with isopropanol. Biological and technical replicates for each population were hybridised to NIA Mouse 44K Microarray v2.3 (whole genome 60 mer oligonucleotide probe; manufactured by Agilent Technologies, #014951) [36]. Fluorescently labelled microarray targets were prepared from 2.5¼ µg aliquots of total RNA samples using a Low RNA Input Fluorescent Linear Amplification Kit (Agilent). A reference target (Cy5-CTP-labeled) was produced from Stratagene Universal Mouse Reference RNA (UMR), and all other targets were labelled with Cy3-CTP. Targets were purified using an RNeasy Mini Kit (Qiagen) according to the manufacturer's protocol and quantified on a NanoDrop scanning spectrophotometer (NanoDrop Technologies). All hybridizations were carried out by combining a Cy3-CTP-labeled experimental target and a Cy5-CTP-labeled UMR target. Microarrays were hybridized and washed according to Agilent protocol (G4140-90030; Agilent 60 mer oligonucleotide microarray processing protocol—SSC Wash, v1.0). Slides were scanned on an Agilent DNA Microarray Scanner, using standard settings, including automatic PMT adjustment.

Pairwise comparisons were performed using standard statistical conditions (FDR <0.05, >1.5-fold expression levels) to unveil genes up-regulated or down-regulated between the populations. Log intensity plots for each gene were created to find pattern matches between those of similar tissue origin.

## Supporting Information

Figure S1 **Karyotypic analysis of HV clones.** Following removal of the selection cassette from the HV cell line, chromosome spreads were prepared from semi-confluent cultures of three Ganc^R^ HV clones for karyotype analysis. Forty chromosomes were observed for each clone.
(2.12 MB TIF)

Figure S2 **Venus expression is up-regulated in ES cell differentiation toward anterior definitive endoderm (ADE).** (A) Schematic of ES cell differentiation toward ADE. HV clones were differentiated in aggregation culture in the presence of activin under conditions designed to promote anterior endoderm differentiation and Hex expression. (B) Venus transgene is expressed in ES cell-derived ADE. Under the conditions diagrammed in (A), the Hex Redstar (HexRS) reporter gives a robust readout of anterior endoderm-specific Hex expression. Parental R26BirA cells were included as a control. Each line was cultured in the presence (+) or absence of activin. At day 7, when endodermal gene expression is optimal, cultures were harvested and analyzed by flow cytometry. (C) Venus expression occurs with the same kinetics as induction of ADE markers. RNA from differentiating ES cell cultures was analyzed for expression of the endodermal markers Hex and Cerberus. Quantitative PCR using the UPL system was carried out to measure the expression levels. Hex and Cerberus levels were normalised to TBP levels for each sample. Normalised levels are related to the undifferentiated R26 BirA sample for each PCR.
(1.12 MB TIF)

Figure S3 **Common microarray signatures among early lineage markers.** Plots are shown comparing mean log intensity values for individual genes among the four populations. Error bars represent standard deviation between expression levels in independent clones of HV ES cells.
(3.61 MB TIF)

Figure S4 **Significant gene expression changes in HV ES cell culture.** Pair-wise comparisons (FDR <0.05, >1.5-fold expression levels) were performed between the following populations of cells to reveal non-redundant, significant changes in gene expression. (A) V^+^S^+^ versus V^−^S^+^, 139 genes up and 123 genes down. (B) V^−^S^−^ versus V^−^S^+^, 30 genes up and 1 gene down. (C) V^+^S^−^ versus V^−^S^+^, 1,636 genes up and 539 genes down. (D) V^+^S^−^ versus V^−^S^−^, 1,520 genes up and 617 genes down. (E) V^+^S^−^ versus V^+^S^+^, 92 genes up and 25 genes down.
(9.83 MB TIF)

Figure S5 **Chimera and contribution potential analysis of Venus positive and negative subpopulations.** A schematic illustration of the experiment is depicted in the top panel. HV cells cultured under self-renewing conditions were subjected to flow cytometry to separate Venus positive and negative ES cell subpopulations and injected into Rosa26 LacZ expressing blastocysts within 1 h of purification. As the host embryo was Rosa26 LacZ, strong LacZ-expressing, blue embryos represent low or no contribution chimeras, whereas faint blue or white embryos represent high levels of ES cell contribution. Representative embryos derived from each fraction are shown together with transverse sections. These are typical of the embryos scored to produce the data in Table S4.
(6.27 MB DOC)

Table S1 **Hierarchical clustering of 2,169 differentially expressed genes among the four fractions, V^−^S^+^, V^+^S^+^, V^−^S^−^, V^+^S^−^.** Differential expression corresponds to FDR <0.05 in ANOVA. Expression intensity is log-transformed (log10), and then centred by subtracting the average, which is shown in a separate column.
(0.67 MB XLS)

Table S2 **Gene Ontology (GO) terms over-represented among genes overexpressed in the V^+^S^−^ fraction compared to V^−^S^−^.** The set of genes was identified using criteria: FDR ≤0.05, change ≥1.5-fold. Only significant GO categories are shown (FDR ≤0.05, *N* members ≥5).
(0.13 MB XLS)

Table S3 **Gene ontology (GO) terms over-represented among genes overexpressed in the V^+^S^+^ fraction compared to V^−^S^+^.** The set of genes was identified using criteria: FDR ≤0.05, change ≥1.5-fold. Only significant GO categories are shown (FDR <0.05, *N* members ≥5).
(0.02 MB XLS)

Table S4 **Assessment of chimera contribution by cells from the V^+^S^+^ and V^−^S^+^ fractions at 9.5 dpc.** The table shows the numbers of resultant embryos scored as low-, medium-, and high-contribution chimeras following the injection of fractionated HV cells into Rosa26 LacZ blastocysts. Cells were fractionated based on Venus and SSEA1 expression by flow cytometry. Examples of typical chimeras are shown in Figure S5.
(0.24 MB DOC)

## Abbreviations

AVE: anterior visceral endoderm
β-gal: β-galactosidase
*dpc*: *days post coitum*
EBs: embryoid bodies
ECATs: ES cell specific transcripts
ES cell: embryonic stem cell
FDR: false discovery rate
GO: gene ontology
HexRS: Hex RedStar
HV: hex venus
ICM: inner cell mass
IRES: internal ribosomal entry site
PrEc: primitive ectoderm
PrEn: primitive endoderm
VE: visceral endoderm
Xen cells: extra-embryonic endoderm cells
