# Supplementary figures and images for: Functional Heterogeneity of Embryonic Stem Cells Revealed through Translational Amplification of an Early Endodermal Transcript
Source: PLoS Biol. 2010 May 25;8(5):e1000379. doi: 10.1371/journal.pbio.1000379 (PMC2876051; doi:10.1371/journal.pbio.1000379)

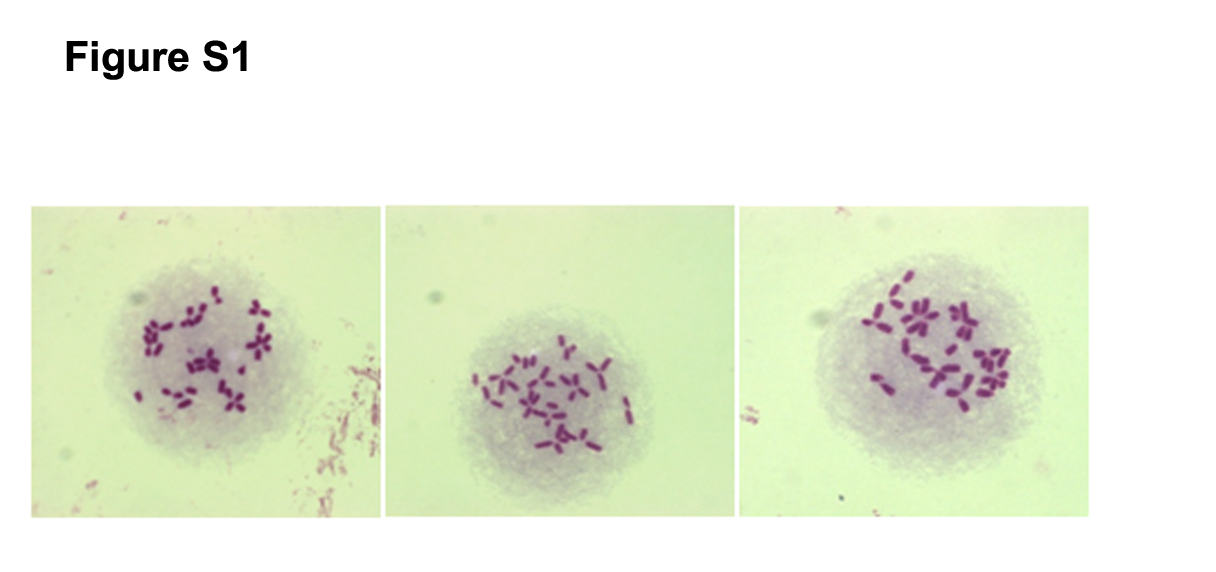

Supplement: Figure S1 — Karyotypic analysis of HV clones. Following removal of the selection cassette from the HV cell line, chromosome spreads were prepared from semi-confluent cultures of three GancR HV clones for karyotype analysis. Forty chromosomes were observed for each clone. (2.12 MB TIF) [file pbio.1000379.s001.tif]

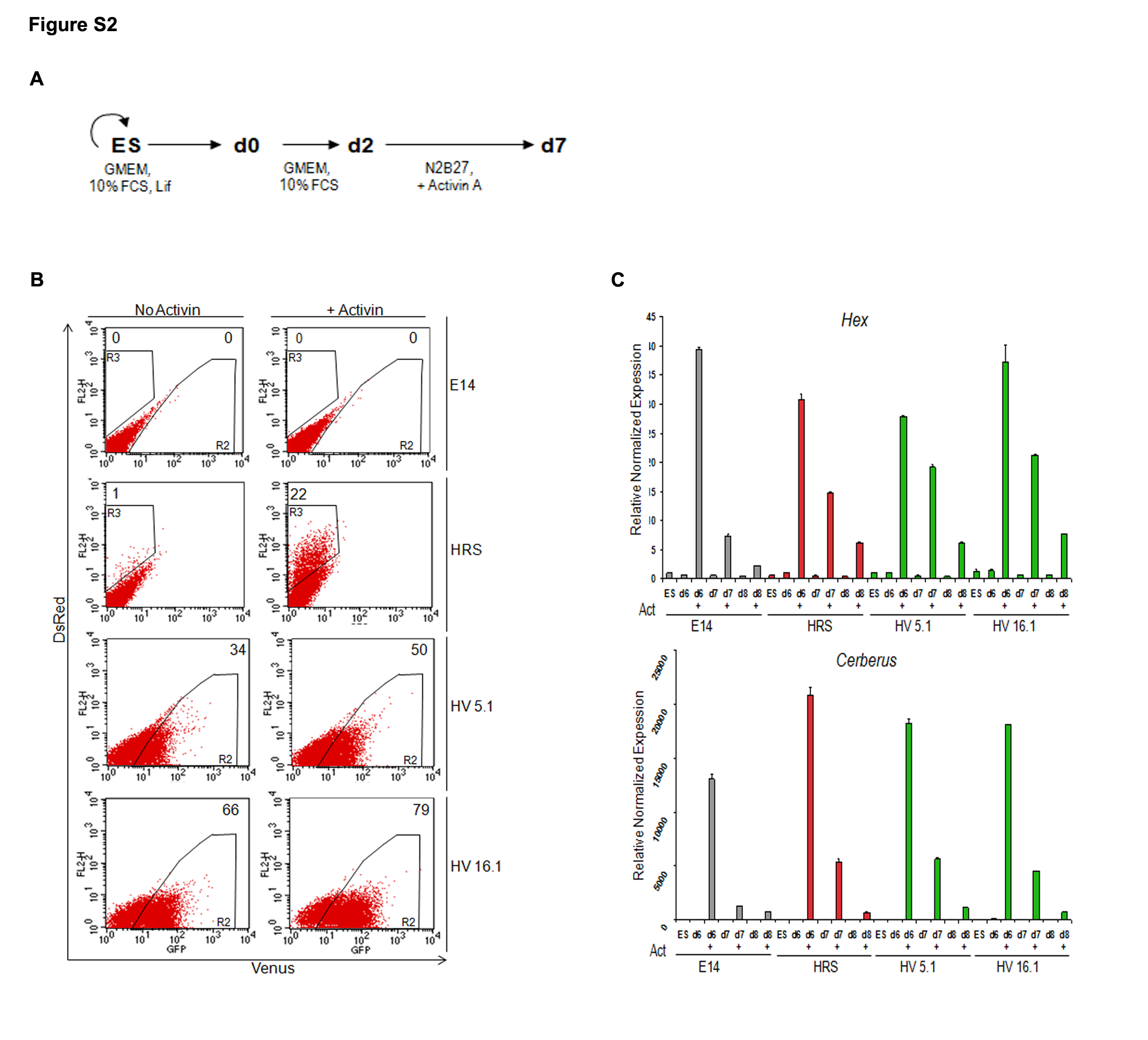

Supplement: Figure S2 — Venus expression is up-regulated in ES cell differentiation toward anterior definitive endoderm (ADE). (A) Schematic of ES cell differentiation toward ADE. HV clones were differentiated in aggregation culture in the presence of activin under conditions designed to promote anterior endoderm differentiation and Hex expression. (B) Venus transgene is expressed in ES cell-derived ADE. Under the conditions diagrammed in (A), the Hex Redstar (HexRS) reporter gives a robust readout of anterior endoderm-specific Hex expression. Parental R26BirA cells were included as a control. Each line was cultured in the presence (+) or absence of activin. At day 7, when endodermal gene expression is optimal, cultures were harvested and analyzed by flow cytometry. (C) Venus expression occurs with the same kinetics as induction of ADE markers. RNA from differentiating ES cell cultures was analyzed for expression of the endodermal markers Hex and Cerberus. Quantitative PCR using the UPL system was carried out to measure the expression levels. Hex and Cerberus levels were normalised to TBP levels for each sample. Normalised levels are related to the undifferentiated R26 BirA sample for each PCR. (1.12 MB TIF) [file pbio.1000379.s002.tif]

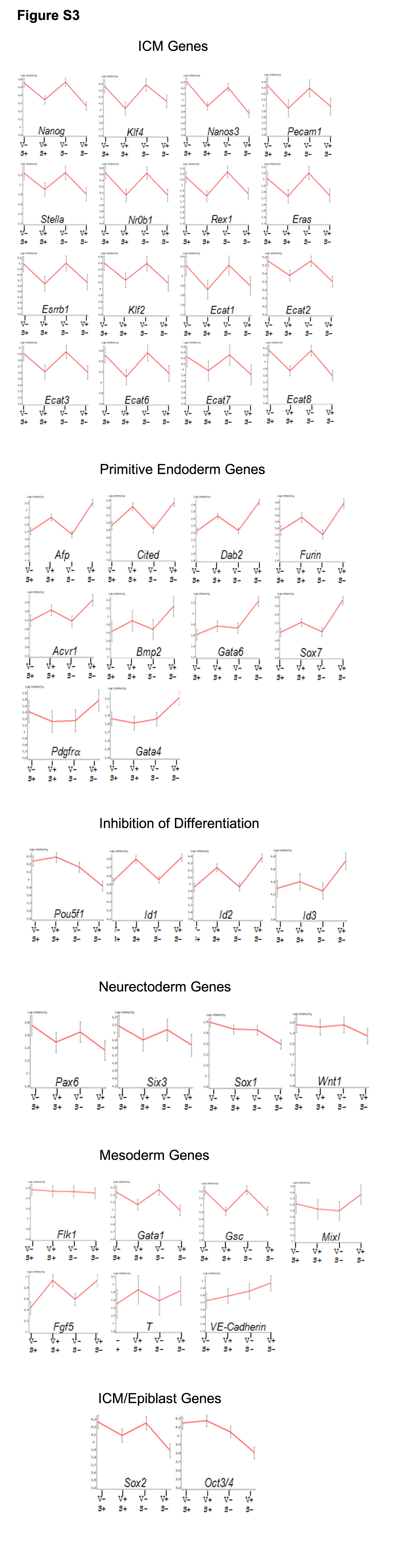

Supplement: Figure S3 — Common microarray signatures among early lineage markers. Plots are shown comparing mean log intensity values for individual genes among the four populations. Error bars represent standard deviation between expression levels in independent clones of HV ES cells. (3.61 MB TIF) [file pbio.1000379.s003.tif]

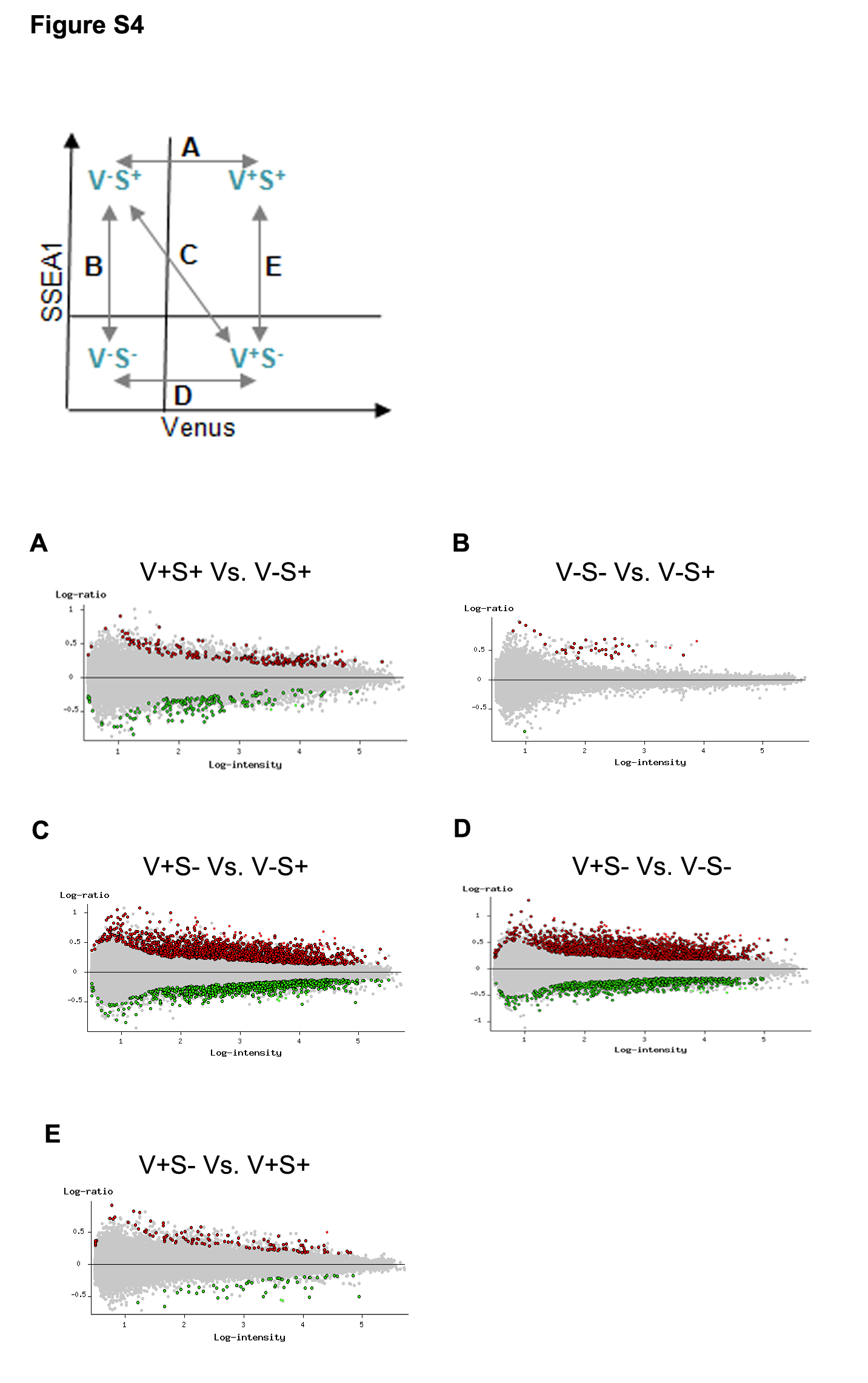

Supplement: Figure S4 — Significant gene expression changes in HV ES cell culture. Pair-wise comparisons (FDR <0.05, >1.5-fold expression levels) were performed between the following populations of cells to reveal non-redundant, significant changes in gene expression. (A) V+S+ versus V−S+, 139 genes up and 123 genes down. (B) V−S− versus V−S+, 30 genes up and 1 gene down. (C) V+S− versus V−S+, 1,636 genes up and 539 genes down. (D) V+S− versus V−S−, 1,520 genes up and 617 genes down. (E) V+S− versus V+S+, 92 genes up and 25 genes down. (9.83 MB TIF) [file pbio.1000379.s004.tif]

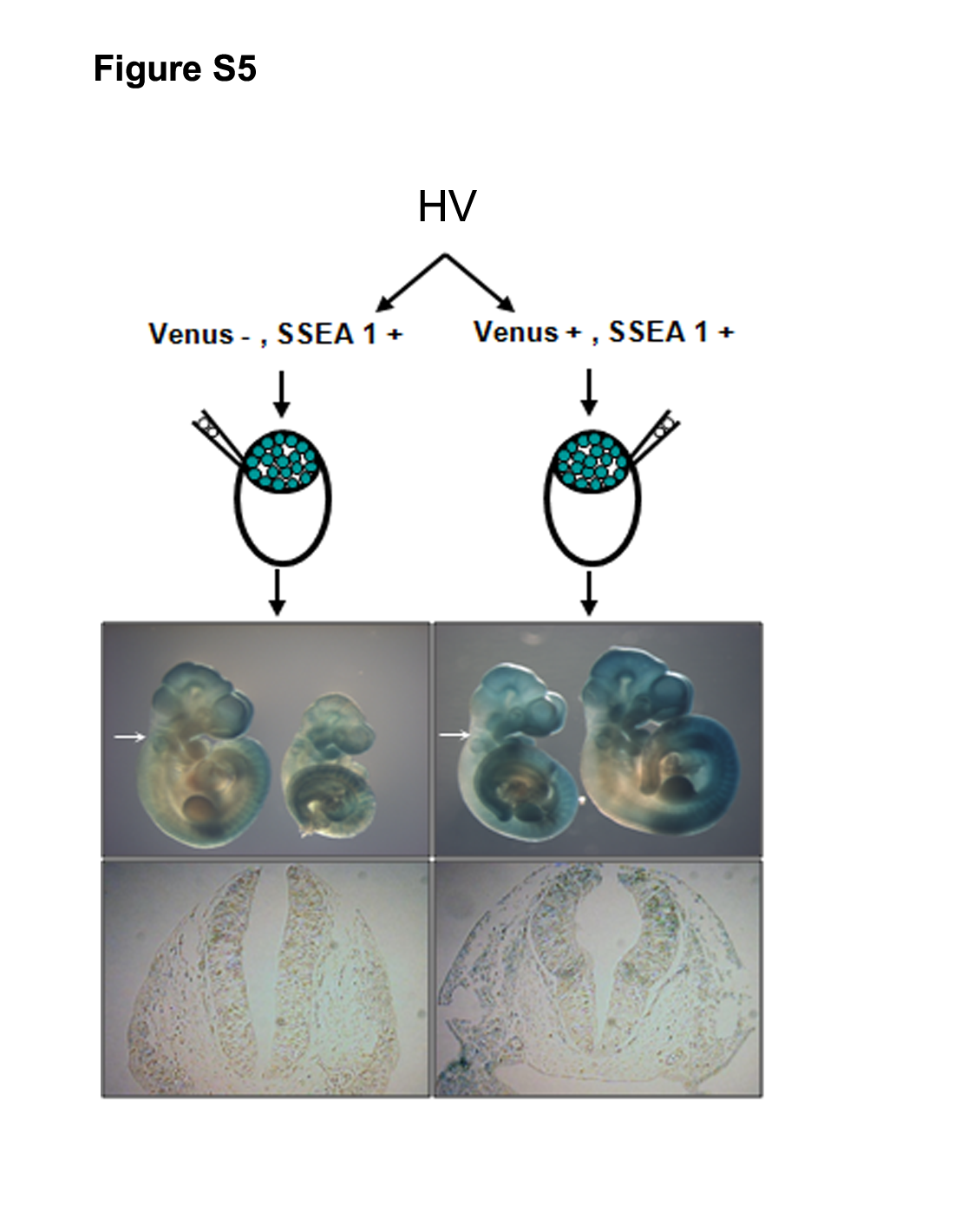

Supplement: Figure S5 — Chimera and contribution potential analysis of Venus positive and negative subpopulations. A schematic illustration of the experiment is depicted in the top panel. HV cells cultured under self-renewing conditions were subjected to flow cytometry to separate Venus positive and negative ES cell subpopulations and injected into Rosa26 LacZ expressing blastocysts within 1 h of purification. As the host embryo was Rosa26 LacZ, strong LacZ-expressing, blue embryos represent low or no contribution chimeras, whereas faint blue or white embryos represent high levels of ES cell contribution. Representative embryos derived from each fraction are shown together with transverse sections. These are typical of the embryos scored to produce the data in Table S4. (6.27 MB DOC) [file pbio.1000379.s005.tif]
