# Supplementary material for: Functional Heterogeneity of Embryonic Stem Cells Revealed through Translational Amplification of an Early Endodermal Transcript
Source: PLoS Biol. 2010 May 25;8(5):e1000379. doi: 10.1371/journal.pbio.1000379 (PMC2876051; doi:10.1371/journal.pbio.1000379)
Supplement: Table S4 — Assessment of chimera contribution by cells from the V+S+ and V−S+ fractions at 9.5 dpc. The table shows the numbers of resultant embryos scored as low-, medium-, and high-contribution chimeras following the injection of fractionated HV cells into Rosa26 LacZ blastocysts. Cells were fractionated based on Venus and SSEA1 expression by flow cytometry. Examples of typical chimeras are shown in Figure S5. (0.24 MB DOC) [file pbio.1000379.s009.doc]

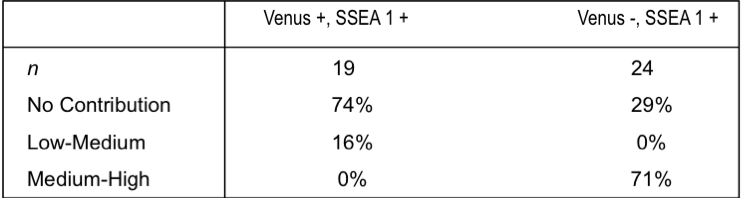


**Supplementary Table 4**. Assessment of chimera contribution of V^+^S^+^ and V^-^S^+^ cells from the HV cell line at 9.5 dpc. Table shows the numbers of resultant embryos from injection into ROSA26 LacZ blastocysts following fractionation based on Venus and SSEA1 expression by flow cytometry.
